# Editorial: Novel chemical, microbiological and physical approaches in food safety control

**DOI:** 10.3389/fnut.2022.1060480

**Published:** 2022-11-02

**Authors:** Marco Iammarino, Sara Panseri, Gulhan Unlu, Giuliana Marchesani, Antonio Bevilacqua

**Affiliations:** ^1^Department of Chemistry, Istituto Zooprofilattico Sperimentale della Puglia e della Basilicata, Foggia, Italy; ^2^Department of Veterinary Medicine and Animal Sciences, University of Milan, Milan, Italy; ^3^Department of Animal, Veterinary and Food Sciences, University of Idaho, Moscow, ID, United States; ^4^Department of Agriculture, Food, Natural Resources and Engineering, University of Foggia, Foggia, Italy

**Keywords:** chemical analysis, food safety, microbiological analysis, physical analysis, novel approaches

Food safety represents a challenge worldwide and despite the increasing advances in food science and technology, the WHO reports that at least one in ten people worldwide fall ill from contaminated food (1). Moreover, food safety is also a challenge because some treatments, although assuring the achievement of food safety objectives, could cause a detrimental effect on food quality. Generally, issues on food safety arise from pathogen growth, fungal contamination of processed foods and raw materials, spoiling microorganisms exerting a detrimental quality on food, unsuccessful approaches leading to an incomplete inactivation of both pathogenic and spoiling microorganisms, problems with detection procedures, while the use of some conventional and unconventional approaches could solve some of these issues, e.g., bio-preservation with probiotic microorganisms, including yeasts, and non-thermal treatments.

Another significant emerging aspect that requires further research in food safety is the simultaneous presence of different contaminants (both microbiological and chemical) in the same food, the so-called “cocktail effect”. Large amounts of data are needed for these evaluations, since the huge number of interactions among different contaminants, which maybe hypothesized, have to be statistically evaluated before confirming an effective risk. Moreover, the range of food to take into account within these studies is very wide. In order to obtain comprehensive datasets, new approaches are needed. These approaches, composed of new analytical procedures, microbiological protocols and chemical/physical determinations, should allow the quick and economic obtainment of many parameters, possibly respecting the environment, in the “green chemistry” perspective (2, 3).

During last decade, the use of gas and liquid chromatography coupled to mass spectrometry has allowed the development of innovative methods able to quantify a large number of food contaminants, using the same sample preparation and chromatographic run. Applications were mainly focused on the determinations of pesticides, veterinary drugs and environmental contaminants in food, but many other employments are under study (metabolomics, protein fingerprint in microbiology, dark matter, etc.). These recent advances are very useful in the context of food inspection, since these novel approaches provide a lot of information in a brief time. A special focus should also be placed on the advent of nanotechnologies in the food safety sector, with several applications in food packaging, shelf-life enhancement, analytical devices, etc. Regarding microbiology aspects, the development of rapid and robust methods for the detection/isolation of the most representative foodborne pathogens (i.e., O157:H7, non-O157 STEC, human noroviruses, etc.) represents a constant challenge in food safety.

The main focus of this Research Topic is to showcase the latest advances in development of new procedures, approaches and technologies for the determination of chemical and microbiological contaminants in food, with a special focus on emerging concerns in food safety. All these lines were addressed in this Research Topic, through the selection of high-quality scientific articles dedicated to food chemistry and microbiology, with a team composed of researchers from various countries (Australia, South Korea, China, India, Italy, Spain, and USA).

A brief overview of all articles collected in this Research Topic is reported below.

The Review prepared by Yemmireddy et al. describes the effects of ultraviolet light treatment on microbiological safety of fresh and fresh-cut fruits and vegetables, since these products are often associated with foodborne illness outbreaks. The authors underlined that the use of UV irradiation on post-harvest processing is limited because of the complexity of food matrices. However, they also reported several studies which demonstrate the high potential of using UV irradiation in combination with other treatments to get similar efficacy to chemical sanitizers.

Fernandez-Pacheco et al. addressed the potential of yeasts as probiotic microorganisms, with a focus also on their safety traits for human health (biogenic amines and other enzymatic traits), stressing the fact the use of probiotic microorganisms is an interesting line for food safety, but they also pose some issues for health, which should be properly addressed.

Yu et al. used Illumina-Based Analysis Yields to study the fungal community of processed products of Crataegi Fructus, a popular medicinal and edible herb in China. The authors identified 115 species. Ascomycota was dominant at the phylum level, while *Dothideomycetes, Pleosporaceae*, and *Alternaria* were dominant at the class, family, and genus levels, respectively. The study provides reliable references for the prevention and control of fungal contamination of Crataegi Fructus.

Dong et al. investigated the Maillard Reaction relating to the possible effects of microwave baking conditions. More in depth, the influence of microwave power and time on the formation of thermal processing hazards (TPHs) and their precursors was studied in microwave-baked biscuits, by means of liquid chromatography techniques. The authors concluded that acrylamide, 5-hydroxymethylfurfural, methylglyoxal, and 3-deoxyglucosone concentrations significantly increase when prolonging the microwave time from 2 to 3 min, and microwave power from 440 to 520 W. The authors also verified that microwave baking allows decreasing the levels of these 4 compounds up to 61.3% with respect to traditional baking (190°C for 7 min).

Bevilacqua et al. focused on the challenge of sub-lethal treatments and how they could pose some safety concerns mainly when non-thermal treatments are used: after a sub-lethal treatment, in fact, injured microorganisms do not die and after a period they could restore their active metabolism and grow again in foods.

Wang et al. described an analytical method based on kompetitive allele specific PCR (KASP) technology and GC-MS/MS for the detection of adulteration and pesticides in Chinese Patent Medicine Qipi Pill. The authors ascertained the presence of 4 pesticides (pentachloronitrobenzene, hexachlorocyclohexane, aldrin and dichlorodiphenyltrichloroethane). Moreover, the main herbal ingredient *Panax ginseng* was partially and completely substituted by *P. quinquefolius*, in 1 and 2 samples out of 12 analyzed, respectively, confirming the need of quality controls for such products.

Zhao et al. developed a metabolomics approach, based on ultra-high-performance liquid chromatography–tandem mass spectrometry combined with multivariate statistical analysis, for geographical origin discrimination of agricultural products. Overall, 159 metabolites were identified and used for discriminating products from Pinggu area (China) from other adjacent regions, successfully.

Finally, the Opinion by Mahato et al. describes several characteristics of nanoencapsulation for agri-food applications with particular focus on associated health and environmental concerns. These authors highlighted the urgency to increase the general knowledge about nanoparticles, as well as all pros/cons related to both beneficial applications and possible associated risk for agri-food applications.

All articles of this Research Topic open a window and new frontiers on food safety and on the challenges that chemists and microbiologists all over the world have to contend with. We hope that readers could find in this Research Topic of Articles new details and information to expand their knowledge and increase their curiosity.

## Author contributions

All authors contributed to the article and approved the submitted version.

## Conflict of interest

The authors declare that the research was conducted in the absence of any commercial or financial relationships that could be construed as a potential conflict of interest.

## Publisher's note

All claims expressed in this article are solely those of the authors and do not necessarily represent those of their affiliated organizations, or those of the publisher, the editors and the reviewers. Any product that may be evaluated in this article, or claim that may be made by its manufacturer, is not guaranteed or endorsed by the publisher.
